# Formulation and Development of Oral Fast-Dissolving Films Loaded with Nanosuspension to Augment Paroxetine Bioavailability: In Vitro Characterization, Ex Vivo Permeation, and Pharmacokinetic Evaluation in Healthy Human Volunteers

**DOI:** 10.3390/pharmaceutics13111869

**Published:** 2021-11-05

**Authors:** Ahmed Hassen Elshafeey, Rania Moataz El-Dahmy

**Affiliations:** 1 Department of Pharmaceutics and Industrial Pharmacy, Faculty of Pharmacy, Cairo University, Cairo 11562, Egypt; 2 Department of Pharmaceutics and Industrial Pharmacy, Faculty of Pharmacy, October 6 University, Central Axis, Cairo 12585, Egypt; rania-moataz@o6u.edu.eg

**Keywords:** paroxetine, nanosuspension, oral fast dissolving film, factorial design, rapid release, ex vivo permeation, bioavailability

## Abstract

Paroxetine (PX) is the most potent serotonin reuptake inhibitor utilized in depression and anxiety treatment. It has drawbacks, such as having a very bitter taste, low water solubility, and undergoing extensive first pass metabolism, leading to poor oral bioavailability (<50%). This work aimed to develop and optimize palatable oral fast-dissolving films (OFDFs) loaded with a paroxetine nanosuspension. A PX nanosuspension was prepared to increase the PX solubility and permeability via the buccal mucosa. The OFDFs could increase PX bioavailability due to their rapid dissolution in saliva, without needing water, and the rapid absorption of the loaded drug through the buccal mucosa, thus decreasing the PX metabolism in the liver. OFDFs also offer better convenience to patients with mental illness, as well as pediatric, elderly, and developmentally disabled patients. The PX nanosuspension was characterized by particle size, poly dispersity index, and zeta potential. Twelve OFDFs were formulated using a solvent casting technique. A 2^2^ × 3^1^ full factorial design was applied to choose the optimized OFDF, utilizing Design-Expert^®^ software (Stat-Ease Inc., Minneapolis, MN, USA). The optimized OFDF (F1) had a 3.89 ± 0.19 Mpa tensile strength, 53.08 ± 1.28% elongation%, 8.12 ± 0.13 MPa Young’s modulus, 17.09 ± 1.30 s disintegration time, and 96.02 ± 3.46% PX dissolved after 10 min. This optimized OFDF was subjected to in vitro dissolution, ex vivo permeation, stability, and palatability studies. The permeation study, using chicken buccal pouch, revealed increased drug permeation from the optimized OFDF; with a more than three-fold increase in permeation over the pure drug. The relative bioavailability of the optimized OFDF in comparison with the market tablet was estimated clinically in healthy human volunteers and was found to be 178.43%. These findings confirmed the success of the OFDFs loaded with PX nanosuspension for increasing PX bioavailability.

## 1. Introduction

Paroxetine (PX) is a phenylpiperidine compound and is the most selective and potent serotonin reuptake inhibitor. Additionally, it blocks muscarinic acetylcholine receptors and is even more effective than maprotiline or desipramine. PX is used to treat anxiety, depression, panic, obsessive-compulsive disorders, and various psychiatric disorders [1]. Although PX is absorbed well from the gastrointestinal tract, it has drawbacks such as a very bitter taste, low water solubility, and undergoing extensive metabolism in the liver, resulting in low oral bioavailability (about 30–50%) [2]. Some efforts have been made to increase PX bioavailability by developing PX in the form of intranasal nanoemulsions [2], transdermal liposomes [3], and as buccoadhesive bi-layer tablets [4]. Nanosuspension systems have many advantages over other drug delivery systems because of their efficient solubility and bioavailability enhancement of almost all low-soluble drugs and because of not utilizing the excipients utilized in other solubility enhancement systems [5]. Successes with nanosuspension formulations in enhancing the bioavailability of many drugs with poor water solubility have been previously reported [6]. However, nanosuspension solidification is always preferred, to improve their physical stability. Different techniques of solidification were utilized, such as vacuum drying, spray-drying, and freeze-drying [7]. However, the drug nanoparticles can irreversibly aggregate in case of an improper drying process, resulting in reducing the dissolution rate. Therefore, loading drug nanosuspensions on films could be the method of choice, because of its process efficiency and simplicity.

This work aimed to develop and optimize novel palatable oral fast dissolving films (OFDFs) loaded with a paroxetine nanosuspension, to improve PX oral bioavailability by decreasing its broad metabolism in the liver and increasing PX solubility and permeability through the buccal mucosa. OFDFs are palatable, robust, and unique delivery systems that offer several advantages over the other conventional dosage forms available [8]. They offer increased compliance by patients with mental illness, as well as by elderly, pediatric, and developmentally disabled patients. They also offer accurate dosing in a portable and convenient format, without the need for measuring devices or water [9]. OFDFs are very thin oral strips that rapidly hydrate and adhere onto the application site. They are dissolved instantly, within 1 min, by the saliva when placed on any oral mucosal tissue, allowing rapid drug absorption through the oral mucosa (pre-gastric absorption) and an early action onset [10]. Furthermore, they offer significantly greater drug bioavailability than the conventional dosage methods because of the pre-gastric absorption of the drug in the buccal cavity, which allows avoiding the first-pass effect, and because even if part of the drug released from the OFDF is swallowed, it will still enter the gastrointestinal tract either dissolved or suspended in the saliva. Thus, it will be present in a readily bioavailability form [11]. Moreover, it is worth mentioning that the oral mucosa is highly vascularized, which helps in rapidly achieving the drug therapeutic serum concentrations [12]. Considering these facts, it is very attractive to use the dual advantages of both, OFDFs loaded with drug nanosuspension, and buccal administration, to support PX indication for the treatment of depression and anxiety.

A PX nanosuspension, prepared using a solvent–antisolvent precipitation method, were loaded into OFDFs prepared using a solvent casting method. In preparing the OFDFs, pectin and carboxy methyl cellulose polymers were used to obtain the desired film toughness, to avoid any damages during transportation or handling [13]. Three types of plasticizers (glycerol, propylene glycol, and polyethylene glycol 400) were incorporated, to impart a certain degree of flexibility to the OFDFs; where these plasticizers convert the used polymer from a hard stiffy glassy state to soft rubbery state by decreasing the polymer’s glass transition temperature [14]. Tween 80 was incorporated as a wetting agent to accelerate the disintegration of the prepared OFDFs within seconds, releasing the loaded drug rapidly [15,16]. Moreover, it was used as a permeation enhancer to increase the PX penetration through the buccal mucosa, because of its non-ionic nature, high HLB value, and low critical micelle concentration [17]. A full factorial design (2^2^ × 3^1^) was implemented, to investigate the formulation variables affecting PX OFDFs properties, utilizing Design-Expert^®^ software. All the prepared OFDFs were investigated by examining their physical characteristics, mechanical characteristics, and dissolution profiles. Comparative dissolution, ex vivo permeation, and a stability study were performed for the optimized PX OFDF. The bioavailability of the optimized PX OFDF was clinically investigated in healthy human volunteers and compared with the market tablet.

## 2. Materials and Method

### 2.1. Materials

Paroxetine (PX) was provided by Glaxosmithkline Beecham, England. Pectin was purchased from Alpha Chemika (Mumbai, India). Carboxymethyl cellulose (CMC) was provided by Dow Wolff Cellulosics (Bomlitz, Germany). Poloxamer 188, citric acid, menthol, and Tween 80 were provided by Sigma Aldrich Co. (St. Louis, MO, USA). Polyethylene glycol 400 (PEG 400), glycerol, and Propylene glycol (PG) were obtained from Prolabo Co. (Paris, France). Sucralose was from El- Gomhouria Chemical Co. (Cairo, Egypt). Paroxetine^®^ 25 mg oral market tablets were from EVA Pharma Co., (Cairo, Egypt). The rest of the solvents and chemicals were of analytical grade.

### 2.2. Preparation of Paroxetine Nanosuspension

Paroxetine nanosuspension was formulated using a solvent–antisolvent precipitation technique [18]. In brief, 250 mg of PX was dissolved in a suitable volume of organic solvent (ethanol) using a magnetic stirrer (SCHOTT, Germany). The anti-solvent phase was prepared by dissolving poloxamer 188 (2% *w*/*v*), as a stabilizer, in 5 mL distilled water using a magnetic stirrer. Then, the organic solvent was injected using a syringe into the aqueous phase under continuous stirring. After precipitation of PX nanoparticles, stirring was continued for a further 2 h to assure complete evaporation of the organic solvent. Then, the resulting nanosuspension was subjected to ultrasonication (CPX 130, Cole Parmar) for 10 min to further reduce the PX particle size.

### 2.3. Assessment of Particle Size, Poly Dispersity Index, and Zeta Potential

Particle size (PS), poly dispersity index (PDI), and zeta potential (ZP) were detected utilizing the dynamic light scattering technique (Nano ZS-90, Malvern Zetasizer, Worcestershire, UK) [15]. Prior to starting the analysis, the PX nanosuspension was first diluted with an adequate amount of distilled water with sonication for 1 min. Measurements were performed three times and the mean was calculated.

### 2.4. Differential Scanning Calorimetry (DSC)

Paroxetine compatibility with both polymers (pectin and CMC), used to prepare the OFDFs, was investigated using a DSC (DSC-60; Shimadzu Corp., Kyoto, Japan) calibrated with indium. PX and physical mixtures of PX with each of the used polymers (in a ratio 1:1) were assessed for any possible interactions. Then, 3 mg from each sample was placed in aluminum pans. Each pan was heated from 20 to 200 °C at a rate of 10 °C/min under a nitrogen atmosphere [19].

### 2.5. Oral Fast Dissolving Films (OFDFs) Loaded with PX Nanosuspension Preparations

OFDFs containing 25 mg PX were prepared using a solvent casting technique [20,21]. Briefly, an precisely weighed quantity of the polymers (pectin or CMC) was added to 5 mL double distilled water and mixed using a magnetic stirrer (Wheaton, Rc-2, Kyoto, Japan) for 1 h. Then, tween 80 was added to that solution, with continuous stirring for 10 min. After, sucralose, citric acid, and menthol were added and stirred till the complete dissolving of all ingredients. After that, a plasticizer (Glycerol, PG, or PEG 400) was added and continually stirred for 1 h till the solution became clear. Then, the previously prepared PX nanosuspension was added to the mixture with continuous mixing for 1 h, till the formation of a homogenous mixture. The obtained mixture was left undisturbed and, after becoming totally free from any bubbles, was poured into a glass Petri dish and dried in a hot air oven at 60 °C for the first 2 h and at 40 °C for the following 24 h. The resulting OFDF was peeled from the glass Petri dish and cut into films with a size of 2 × 2 cm^2^, each contained 25 mg PX. Then, they were wrapped, utilizing airtight aluminum foil. Any film with imperfections, cuts or air bubbles was excluded.

### 2.6. Full Factorial Statistical Design

A 2^2^ × 3^1^ full factorial design was implemented to detect the impact of the investigated factors on the PX OFDF’s properties, utilizing Design-Expert^®^ software (Stat-Ease Inc., Minneapolis, MN, USA). The investigated factors were polymer type (X_1_), polymer concentration (X_2_), and plasticizer type (X_3_). The studied responses were tensile strength (Y_1_), elongation % (Y_2_), Young’s modulus (Y_3_), disintegration time (Y_4_), and % PX dissolved after 10 min (Y_5_). Twelve experimental runs were prepared, where each run was performed three times, to determine the repeatability of the results. The detailed composition of the prepared OFDFs is displayed in Table 1. The optimized OFDF was selected based on those showing the highest desirability. The chosen optimized OFDF had the highest tensile strength, elongation %, and % PX dissolved after 10 min, and the lowest Young’s modulus and disintegration time. Finally, the chosen optimized OFDF was characterized by comparing its observed and predicted responses to ensure the model performance accuracy.

### 2.7. Characterization of Paroxetine OFDFs

#### 2.7.1. Average Weight

Each film was weighed individually, utilizing an electronic balance (type AX200, Shimadzu corp., Kyoto, Japan) [22]. Each test was repeated three times.

#### 2.7.2. Film Thickness

The thickness of each OFDF was measured at five different positions, the center and the four corners, utilizing digital Vernier calipers (Mitutoyo, Kawasaki, Japan). This test was repeated three times, and the average ± SD was calculated [8].

#### 2.7.3. Folding Endurance

This test was performed to check the flexibility of each film, needed to provide facile handling and administration. The film was folded or rolled repetitively until it broke. The number of folds that occurred before the breakage was counted and considered the folding endurance value. The folding times (folding endurance value) and the film flexibility were directly proportional [23].

#### 2.7.4. Content Uniformity

This test was implemented to ensure a uniform distribution of PX in each OFDF. Each film was dissolved in a 100-mL volumetric flask containing 10 mL ethanol using a vortex (VM-300, Gemmy Industrial Corp., Taiwan) for five minutes [8]. The sample from the resulted liquid was diluted with an appropriate ethanol volume. PX content was detected utilizing a UV spectrophotometer (UV-1700; Shimadzu, Kyoto, Japan) at 294.3 nm [3]. PX average content ± SD was determined using the following equation [22]:

(1) $$\mathrm{Content}\;\mathrm{uniformity}=\frac{\mathrm{Actual}\;\mathrm{PX}\;\mathrm{amount}\;\mathrm{in}\;\mathrm{OFDFs}}{\mathrm{Theoretical}\;\mathrm{PX}\;\mathrm{amount}\;\mathrm{in}\;\mathrm{OFDFs}}\times 100$$

This test was repeated three times.

#### 2.7.5. Surface pH

Each film was moistened in a closed Petri dish using distilled water (1 mL) and kept for 5 min at 25 °C. The pH of each OFDF was assessed utilizing a pH meter (Jenway 3510, Swedesboro, USA) by touching an electrode with a moistened surface to each OFDF. The test was performed in triplicate, and the mean value was calculated [24].

#### 2.7.6. Moisture Content %

Percentage moisture content is an essential parameter to detect the integrity and the physical stability of formed films [21]. Moisture content %, included the two important parameters of moisture loss % and moisture absorption %.

##### Moisture Loss %

Moisture loss % was assessed by recording the initial weight of each OFDF, then putting these films in a desiccator that contained anhydrous calcium carbonate at room temperature for 72 h [25]. After that, OFDFs were taken out from the desiccator and reweighed. This parameter was determined according to the following equation [21]:

(2) $$\;\mathrm{Moisture}\;\mathrm{loss}\;\%\;=\frac{\mathrm{Initial}\;\mathrm{weight}\;-\;\mathrm{Final}\;\mathrm{weight}\;}{\mathrm{Initial}\;\mathrm{weight}\;}\times 100$$

##### Moisture Absorption %

This parameter was determined by putting pre-weighed films in a desiccator containing potassium chloride saturated solution, to achieve a 80% relative humidity for 24 h, at room temperature. Then, each film was weighed again and the moisture absorption % of each OFDF was assessed using the following equation [14]:

(3) $$\mathrm{Moisture}\;\mathrm{absorption}\;\%\;=\;\frac{\mathrm{Final}\;\mathrm{weight}\;-\;\mathrm{Initial}\;\mathrm{weight}\;}{\mathrm{Initial}\;\mathrm{weight}\;}\times 100$$

#### 2.7.7. Mechanical Characteristics of PX OFDFs

The mechanical characteristics of the OFDFs were measured utilizing a digital tensile testing machine (Lloyd Instruments Ltd., LR 10K, UK). The thickness of each OFDF had previously been detected utilizing a digital Vernier caliper. Each OFDF was precisely cut into a rectangular shape using a metal scalpel blade [26]. The tensile tester was set with an initial grip separation of 25 mm and crosshead speed of 35 mm/minute. Each OFDF was placed vertically between the tensile tester’s two clamps. Each OFDF was pulled apart by the two clamps until breakage [27]. For each OFDF, measurements were performed three times, and the mean value ± SD was determined.

##### Tensile Strength

Tensile strength is the maximum force required to break the films. This was performed to detect the strength of the OFDFs. Tensile strength was expressed as force/unit area or mega Pascal (MPa), which was estimated as follows [28]:

(4) $$\mathrm{Tensile}\;\mathrm{strength}\;=\;\frac{\mathrm{Force}\;\mathrm{at}\;\mathrm{breakage}\;\left(\mathrm{kg}\right)}{\mathrm{Film}\;\mathrm{thickness}\;\left(\mathrm{mm}\right)\times \;\mathrm{film}\;\mathrm{wedth}\;\left(\mathrm{mm}\right)}$$

##### Percent Elongation

The % elongation (strain) was assessed to detect the toughness and stretching of the prepared OFDFs. The stretching and elongation of the film prior to breakage are referred to as the %Elongation or strain. Percent elongation was determined as follows [16]:

(5) $$\;\%\;\mathrm{Elongation}\;=\frac{\mathrm{Increase}\;\mathrm{in}\;\mathrm{the}\;\mathrm{OFDF}\;\mathrm{length}\;}{\mathrm{Initial}\;\mathrm{length}\;\mathrm{of}\;\mathrm{the}\;\mathrm{OFDF}}\times 100$$

##### Young’s Modulus

Young’s modulus, or elastic modulus, was utilized to estimate the elasticity or stiffness of each film. It was calculated from the slope obtained from the linear portion of the stress–strain curve, as shown in this equation [26]:

(6) $$\mathrm{Young}’s\;\mathrm{modulus}\;=\;\frac{\mathrm{Slope}\;}{\mathrm{Thickness}\;\mathrm{of}\;\mathrm{the}\;\mathrm{Film}\;X\;\mathrm{Crosshead}\;\mathrm{speed}}\;\times 100$$

#### 2.7.8. In Vitro Disintegration Time

Each OFDF was placed in a glass Petri dish, then, 10 mL of Sorensen’s phosphate buffer (pH 6.8) was added to the petri dish at 25 °C. The time required to disintegrate or break each OFDF was recorded [27]. For each OFDF, measurements were performed three times, and the mean value was calculated.

#### 2.7.9. In Vitro Dissolution Study

The PX in vitro release from all the prepared OFDFs was evaluated in a USP dissolution apparatus (type II) (Pharm Test, Hainburg, Germany). The dissolution medium was composed of 250 mL Sorensen’s phosphate buffer with pH of 6.8 [29]. Throughout the study, the temperature was adjusted to 37 ± 0.5 °C, and the rotation speed was fixed at 50 rpm. Each PX OFDF was put at the bottom of the dissolution apparatus vessel. Then, 4 mL of sample was withdrawn at 2, 4, 6, 8, 10, 15, 30, 45, and 60 min time intervals. Then, 4 mL of fresh phosphate buffer was replenished to keep a constant medium volume [30,31]. Finally, PX concentration was detected spectrophotometrically in each sample at λ_max_ 294.3 nm [3]. The test was performed three times and the mean % PX dissolved was estimated [32].

### 2.8. Characterization of the Optimized OFDF Loaded with PX Nanosuspension

#### 2.8.1. Re-Dispersion of PX Nanoparticles from the Optimized OFDF

The optimized OFDF (F1) was dispersed in 10 mL deionized water. After 10 min of magnetic stirring, the size of nanoparticles was measured, as explained earlier [15].

#### 2.8.2. Comparative Dissolution Study of Optimized PX OFDF, Drug Powder, and the Market Tablet

A comparative dissolution study between the optimized PX OFDF (F1), drug powder, and the market tablet (Paroxetine^®^ 25 mg) was performed to investigate the success of the optimized OFDF in increasing the dissolution rate [16,27]. The study was implemented using the same conditions as mentioned previously for the in vitro dissolution study. Dissolution T_50%_ and similarity factor (f_2_) were estimated to compare PX release profiles of the optimized PX OFDF (F1), drug powder, and the market tablet.

#### 2.8.3. Ex Vivo Permeation

##### Tissue Preparation

A permeation study was carried out to evaluate the PX permeability across a buccal chicken pouch membrane, to simulate buccal mucosa permeability. After obtaining the membrane from a slaughtered chicken, the underlying connective tissues was separated utilizing forceps. Then, the excised membrane was carefully washed and stored in normal saline at −20 °C. Prior to starting the permeation studies, the buccal mucosa was hydrated with a simulated saliva solution (pH 6.8), prepared by dissolving 0.2 g KH_2_PO_4_, 2.4 g Na_2_HPO_4_, and 8.0 g NaCl in distilled water (1000 mL) [33]. Then the buccal mucosa was thawed till it reached room temperature, prior to use.

##### Ex Vivo Permeation Testing

A USP dissolution apparatus (type I) (Pharm Test, Hainburg, Germany) was used to compare the PX permeation of both the optimized OFDF (F1) and the equivalent drug powder [15,16]. The buccal mucosa was firmly fixed to a glass cylinder (internal diameter 1 cm), with one side as the donor chamber. The optimized film (F1) was put in close contact with the surface of the membrane mucosa at one end of the glass tube. Then 1 mL of phosphate buffer with a pH of 6.8 was added to the glass tube, which was then attached to the dissolution apparatus shaft [16]. The same study was carried out using an equivalent amount of the pure drug, which was put inside the donor chamber and hydrated using 1 mL of pH 6.8 phosphate buffer. An ex vivo permeation study was performed utilizing the same method used in the in vitro release study. Whereby, 4 mL samples were withdrawn at time intervals of 5, 10, 15, 20, 25, 30, 45, and 60 min [15,16]. PX concentration in the gathered samples was analyzed utilizing HPLC (Shimadzu, Tokyo, Japan). Where, a C18 column (5 μm, 150 × 4.6 mm) (Thermo Fisher Scientific, NJ, USA) was the utilized column, and the used mobile phase was composed of 0.04 M phosphate buffer/acetonitrile (65:35), with a 1 mL/min flow rate for all separations. Permeated PX was detected at 294.3 nm [33]. The lower limit of quantification (LLOQ), accuracy, and linearity were estimated. The permeation study was implemented in triplicate and the mean value was recorded.

##### Permeation Parameter Calculation

The cumulative amount of PX permeated per cm^2^ was plotted versus time.

The flux (*J*) was assessed utilizing the following equation [34]:

(7) $$\;J=\frac{\mathrm{Amount}\;\mathrm{of}\;\mathrm{PX}\;\mathrm{permeated}\;\mathrm{from}\;\mathrm{the}\;\mathrm{optimized}\;\mathrm{film}}{\mathrm{Time}\;\times \;\mathrm{memberane}\;\mathrm{area}}$$

Moreover, the permeation enhancement ratio (ER) was estimated utilizing the following equation [35]:

(8) $$\;\mathrm{ER}=\frac{J\;\mathrm{of}\;\mathrm{the}\;\mathrm{optimized}\;\mathrm{film}}{\;J\;\mathrm{of}\;\mathrm{drug}\;\mathrm{reference}}\times 100$$

##### Statistical Analysis

One-way ANOVA was utilized to statistically detect any significant differences in the obtained results, where the difference was considered significant when the *p*-value < 0.05 [36].

#### 2.8.4. Stability Study

A stability study was implemented with the optimized PX OFDF (F1), according to the guidelines of ICH Q1A (R2) [37]. In this study, the temperature was set at 40 ± 2 °C and the relative humidity was adjusted to 75 ± 5%. Each film was covered with aluminum foil and then put in a desiccator for six months. After half of, and the full, storage period, each film was investigated for any changes in content uniformity, tensile strength, % elongation, Young’s modulus, disintegration time, and % PX dissolved after 10 min. The resulting values were subjected to statistical analysis using Student’s t-test and utilizing SPSS 17.0 software (SPSS Inc., Chicago, IL, USA). Differences were considered significant when the *p*-value was less than 0.05.

### 2.9. In Vivo Clinical Studies

#### 2.9.1. In Vivo Disintegration Time and Palatability Studies

The taste masking of bitter drugs represents a fundamental challenge during the preparation of oral fast dissolving films, as most drugs are unpalatable; influencing patient’s compliance and acceptance. The study was executed according to the Helsinki Declaration for bio-medical research, including human and good clinical practices (GCP) rules [38,39]. The protocol of this study was revised and accepted by the institutional review board of the Genuine Research Center, (Cairo, Egypt). Eight healthy human volunteers participated in this double-blind crossover study. All the enrolled volunteers signed a written informed consent, after telling them about the study purpose, nature, duration, and risks. Moreover, all the volunteers were instructed to maintain their standard dietary condition and normal physical activity for five days prior to intaking the dosage form [40]. The volunteers were administrated the optimized PX OFDF (F1) by putting it in the buccal cavity till its complete disintegration. All the enrolled volunteers were asked to record the texture, disintegration time, acceptance (bitterness taste masking), and aftertaste of both the optimized OFDF (F1) and PX pure powder. The scale of bitterness intensity was as follows: 1 = very bitter, 2 = bitter, 3 = acceptable (no specific feeling), 4 = pleasant, 5 = very pleasant [41]. Both the tested optimized OFDF (F1) and the drug pure powder contained the same quantity (25 mg of) PX.

#### 2.9.2. Pharmacokinetic Evaluation in Healthy Human Volunteers

##### Study Design and Subjects

In this comparative study, six healthy male adult volunteers were enrolled. Whereby, a randomized crossover, two period, single dose design was implemented to assess the pharmacokinetic parameters of the optimized PX OFDF (F1) compared to the market tablet (Paroxetine^®^ 25 mg). Their average body weight and age was 72.4 ± 6.7 kg and 40.6 ± 5.0 years, respectively. All the enrolled volunteers signed a written informed consent after receiving the required information about the nature, purpose, and risks of the study. In addition, all volunteers were asked to stop smoking or administrating any medicines for 2 weeks before starting the study, till its end. This study was executed according to the Helsinki Declaration for bio-medical research, including human and the good clinical practice (GCP) rules [38,39]. The protocol of this study was revised and accepted by the institutional review board of Genuine Research Center (Cairo, Egypt). The approval number is GRC/1/21/R4. A specialized physician was employed to observe all volunteers during the study.

##### Drug Administration and Sample Collection

The participating volunteers were randomly distributed into two equal groups. The first treatment was the optimized PX OFDF (F1), and the second treatment was the market tablet. Both the optimized PX OFDF and the market tablet contained 25 mg paroxetine. A seven day washout period was implemented to separate the two phases from each other. After that, the study was repeated using the same administrated conditions, to assure that each of the six volunteers received both treatments. Prior to the study, all the volunteers fasted overnight (ten hours). The participating volunteers placed the optimized PX OFDF (F1) in their buccal cavity till it completely disintegrated and disappeared in their mouths, followed by receiving 200 mL of water. An equivalent volume of water was utilized to swallow the market tablet. Two hours after intaking the dose, volunteers were allowed to drink water, then after an additional 2 h, they were allowed to eat. After that, the volunteers were given standard breakfast, lunch, and dinner meals, in accordance with a planned time schedule.

For PX analysis, an indwelling cannula was utilized to transfer the withdrawn blood samples (5 mL) to heparinized glass tubes at different time intervals of 0.0, 0.33, 0.66, 1, 1.5, 2, 2.5, 3, 3.5, 4, 4.5, 5, 6, 8, 12, 24, and 48 h after dose intake. The gathered blood samples were centrifuged (Hettich EBA 21 Centrifuge, Germany) at 3500 rpm at 4 °C for 10 min. Finally, the obtained plasma was frozen at −20 °C (Ultra-Low Freezer, Cincinnati, OH, USA) until PX analysis.

##### Sample Preparation

All the frozen human plasma samples were left to reach room temperature. Then, a 0.5 mL aliquot from each sample was mixed with 100 μL paroxetine-D6 maleate (internal standard) (IS). Then, the aliquots were vortexed for 1 min. Next, after the addition of 4 mL of methyl-t-butyl-ether (MTBE), the aliquots were vortexed again for two minutes. Thereafter, the resulting samples underwent centrifugation for ten minutes at room temperature, at a speed of 4000 rpm. A centrifugal vacuum concentrator (Vacufuge^®^ 5301, Germany) was utilized to evaporate the obtained clear supernatant till dryness at 40 °C. Then, 100 μL of mobile phase was utilized to reconstitute the resulting dry residues and an autosampler was used to inject 10 µL of the resulting samples [42].

##### Chromatographic Conditions

The LC-MS/MS method is an accurate, selective, and sensitive method, utilized to analyze Paroxetine human plasma samples. Before the study, this method was developed and validated according to international guidelines [43]. To prepare the stock solution of Paroxetine-D6 (the internal standard), 10 mg was dissolved in methanol, then serial dilutions were prepared using the mobile phase. The utilized pump was an LC-20AD (Shimadzu, Japan). The degasser was a DGU-20AS (Shimadzu, Japan), and the utilized auto-sampler for injection of 10 μL samples was an SIL-20A (Shimadzu, Japan). The used mobile phase was composed of formic acid 0.1% in acetonitrile:water (6:4; *v/v*) and delivered at a flow rate of 0.15 mL/minute using a Luna C18 (Phenomenex, Torrance, CA, USA) (50 × 2 mm, 5µm) column [44]. An API-4000 mass spectrometer (Applied Biosystems, Canada), equipped with a nitrogen generator, was used for the quantitation, with a positive ion polarity mode for both paroxetine and Paroxetine-D6 and adjusted with an ion spray voltage of 5500 V. The ion source parameters of entrance potential, collision exit potential, collision energy, and declustering potential were 10, 10, 29, and 100, respectively, for Paroxetine, and 10, 16, 31, and 131 for Paroxetine-D6. Ion detection was implemented in the multiple reaction monitoring mode (MRM), monitoring the transition of m/z 329.6 precursor ion to m/z 192.3 for Paroxetine and the m/z 336.2 precursor ion to m/z 198.1 for Paroxetine-D6. Analytical data processing was implemented utilizing version 1.6.3 of Analyst^®^ software.

##### Pharmacokinetic and Statistical Analysis

A paroxetine pharmacokinetic analysis was made for each subject using a noncompartmental approach, applying Kinetica^®^ 4.4.1 SPSS 14 software (Thermo Fisher Scientific Inc., Waltham, MA, USA). Paroxetine concentration–time curves were used to obtain the PX peak concentration (C_max_, ng/mL) and the time of PX peak concentration (T_max_, hours). The area under the concentration–time curve from zero to the last analyzed point (AUC_0–48_, ng.h/mL), and from zero to infinity (AUC_0–∞_, ng.h/mL), was estimated using the linear trapezoidal rule. T_1/2_ (hours) was assessed as 0.693/K. All the pharmacokinetic parameters of the two treatments were compared utilizing an ANOVA test. Differences between any two related parameters were considered statistically significant if the *p*-value was ≤0.05.

## 3. Results and Discussion

### 3.1. Particle size, Poly Dispersity Index, and Zeta Potential

PS and PDI are important characterization parameters for nanosuspension evaluation. Both of these parameters have a significant effect on an active ingredient’s stability, solubility, dissolution rate, and bioavailability. The prepared PX nanosuspension had PS and PDI values of 217.09 ± 4.18 nm and 0.46 ± 0.27, respectively. The low value of the PDI indicates the small distribution of the particle sizes, and, hence, the homogeneity of the diameter of particles. This low PDI value decreased the PX concentration gradients in the medium, which aids in inhibiting the occurrence of Ostwald ripening [45]. ZP is an essential parameter for indicating the physical stability of prepared nanosuspensions. The ZP value of the formulated PX nanosuspension was −33.49 ± 2.08 mV. A higher ZP value indicates the presence of a large number of particles with the same charges, leading to electrostatic repulsion between them, which prevents their aggregation and maintains their physical stability [46].

### 3.2. Differential Scanning Calorimetry (DSC)

**Figure 1:**
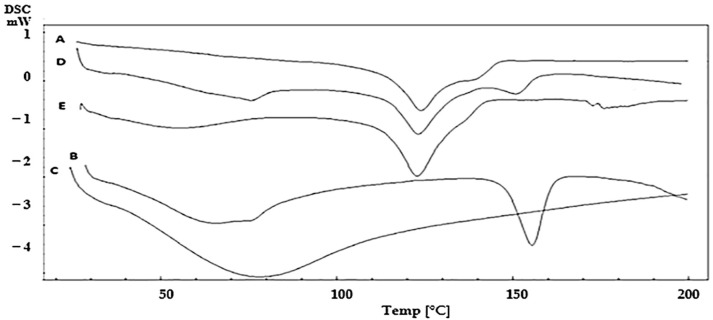
Differential scanning calorimetric (DSC) thermograms of (A) Paroxetine, (B) pectin, (C) CMC, physical mixture of Paroxetine with (D) pectin and (E) CMC.

DSC was utilized to test the PX compatibility with the studied polymers (pectin and CMC). Figure 1 shows the DSC thermograms of PX, pectin, CMC, and the physical mixtures of PX with each polymer, with a ratio (1:1), as this ratio maximizes the possibility of monitoring any interactions [47]. DSC thermograms showed that pure PX had an endothermic peak at 123.75 °C [5]. Pectin showed an endothermic peak at 154 °C, while CMC showed a broad endothermic peak at 78.21 ºC [48,49]. It is worth mentioning that there was no shift in PX peak in the case of both physical mixtures, revealing chemical and physical compatibility.

### 3.3. Preparation of OFDFs Loaded with PX Nanosuspension

A solvent casting method was successfully utilized to prepare the PX OFDFs. The prepared OFDFs were durable and were able to withstand normal handling without any imperfections, cuts, cracks, or air bubbles. Pectin and CMC polymers were successful in obtaining the desired film toughness, to avoid any damages during transportation or handling [13]. Glycerol, PG, and PEG 400, which were used as plasticizers, imparted a certain degree of flexibility to the OFDFs [50]. Citric acid was used as a salivary stimulant to stimulate the saliva secretion in the buccal cavity, thus improving the disintegration of the OFDFs [51]. Sucralose was utilized as a sweetening agent to mask paroxetine’s bitter taste. Whereby, sucralose has 200–600 times more sweetness than sucrose [52]. Menthol was utilized as a flavoring agent to provide a refreshing sensation in the mouth upon administration to the buccal cavity [53].

### 3.4. Full Factorial Design Statistical Analysis

A full factorial (2^2^ × 3^1^) design was implemented using ANOVA because it is beneficial for evaluating the effect of different formulation factors, with their different levels, on the investigated responses. Sum-of-squares (Type II model) was the chosen model. Adequate precision was utilized, to assure that the chosen model was appropriate for navigation of the design space. Table 2 shows that the adequate precision value was >4 in all the responses, which was desirable. For all dependent variables, there was a good harmony between both the predicted and adjusted R^2^ values. Table 2 displays all the investigated factors and the detected responses of the statistical design. The *p*-value of all responses was considered significant (<0.05). The effects of each studied factor on the responses and the graphical response surface plots were obtained utilizing Design expert 8.0.7 software (Stat-Ease Inc., Minneapolis, MN, USA).

### 3.5. Characterization of the Prepared PX OFDFs

#### 3.5.1. Average Weight

Table 3 displays that weight of the different prepared OFDFs varied from 36.86 ± 3.96 mg to 52.61 ± 2.67 mg. These results show the lack of any significant weight variations, which reveals the efficiency of the implemented method and indicates the uniformity of PX distribution.

#### 3.5.2. Films Thickness

Measuring the thickness of all the prepared OFDFs was important, to assure the uniformity of their thickness, and because the dose accuracy is directly related to the film thickness. Generally, an ideal OFDF should exhibit a thickness between 0.05 and 1 mm [8]. Table 3 shows that the thickness of all the prepared OFDFs ranged from (0.11 ± 0.02 mm to 0.23 ± 0.03 mm). The presence of slight variations in films thickness could be attributed to their containing different amounts of polymers. It is worth noting that the thickness of the prepared OFDFs was increased by increasing the amount of the used polymers.

#### 3.5.3. Folding Endurance

The elasticity and flexibility of the prepared OFDFs were important physical characteristics, required for easy handling and the application of the OFDF to the administration site [29]. The folding endurance of all OFDFs ranged from 187 ± 5.71 to >300, as displayed in Table 3. All the prepared OFDFs exhibited good folding endurance values, indicating their good flexibility, possibly due to incorporation of the plasticizers.

#### 3.5.4. Content Uniformity

The PX content was from 89.48 ± 1.09 to 96.68 ± 3.62% of the labeled claim, as shown in Table 3. These results indicate a good drug uniformity in all the prepared OFDFs, assuring that PX was uniformly dispersed in each prepared film.

#### 3.5.5. Surface pH

The pH of each OFDF should be close to the buccal cavity pH (neutral pH) [8]. The pH of all the prepared OFDFs was varied from 6.61 ± 0.38 to 7.01 ± 0.40, as displayed in Table 3. These results assured the absence of any possible irritation to the buccal mucosal lining that could happen due to alkalinity or acidity.

#### 3.5.6. Moisture content %

##### Moisture Loss %

Studying the moisture loss % was undertaken to evaluate the integrity and the physical stability of each OFDF [21]. The moisture loss % of all the prepared OFDFs ranged from 0.93 ± 0.02 to 1.24 ± 0.03%, as shown in Table 3. These results were in an acceptable range, indicating little moisture loss and the good physical integrity and stability of the prepared OFDFs [27]

##### Moisture Absorption %

The moisture absorption % of the films is important, because it influences the friability, mechanical strength, adhesive properties, disintegration, and dissolution behaviors of each film [16]. Table 3 shows that the moisture absorption % of all the prepared OFDFs were from 1.06 ± 0.08% to 8.73 ± 0.38%. It was noticed that the moisture absorption % of the OFDFDs was slightly increased when increasing the polymer concentration. This could have been because of the hydrophilic nature of the used polymers [54]. It was also noticed that the OFDFs made of CMC polymer showed a high water sorption; therefore, they seemed to be not suitable for use at high humidity, which makes the films sticky and unsuitable for this application [13].

#### 3.5.7. Mechanical Characteristics of the OFDFs

##### Tensile Strength

An ideal OFDF should exhibit an adequately high tensile strength value to be able to withstand normal handling. In spite of this, a very high value (very high rigidity) is not desired, because it could retard the drug release from the polymer matrix [29]. The prepared OFDFs had tensile strength values from 1.04 ± 0.11 Mpa to 15.5 ± 0.68 Mpa, as displayed in Table 3. Tensile strength values were analyzed using the following equation:

Tensile strength = 6.39 − 1.72 × X_1_+ 1.25 × X_2_ − 3.04 × X_3_ [1] + 3.73 × X_3_ [2] (9)

where X_1_ is the polymer type, X_2_ is the polymer concentration, and X_3_ is the plasticizer type. X_3_ [1] represents the first plasticizer type (glycerol) and X_3_ [2] represents the second plasticizer type (propylene glycol).

**Figure 2:**
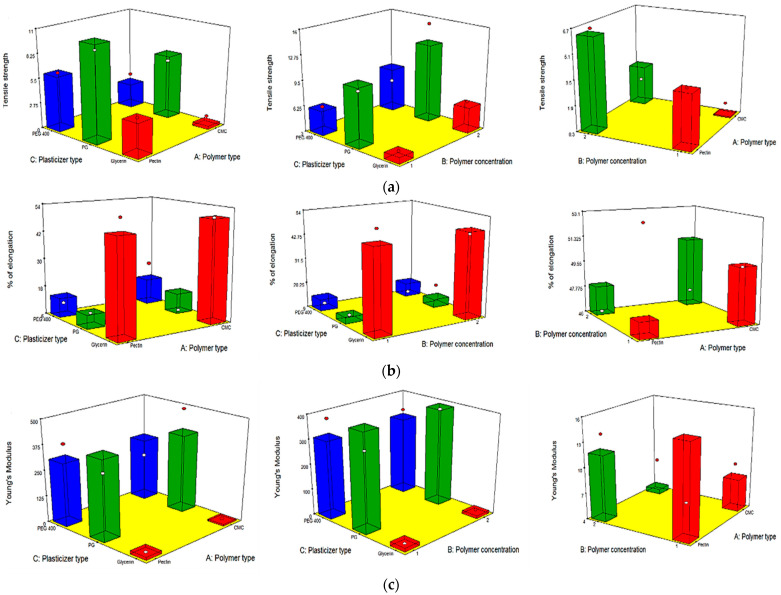
Response 3-D plots for the effect of polymer type (X_1_), polymer concentration (X_2_), and plasticizer type (X_3_) on (**a**) tensile strength, (**b**) elongation %, and (**c**) Young’s modulus of the OFDFs loaded with PX nanosuspension.

Table 3 and Figure 2a show that all the independent variables (X_1_, X_2_, and X_3_) had a significant impact on the tensile strength (rigidity) of all the OFDFs loaded with PX. It was obvious that the tensile strength values were significantly changed when changing the type of polymer used (X_1_). Where OFDFs prepared using pectin polymer showed significantly higher tensile strength values than those prepared with CMC polymer. This could be accredited to the differences in the nature and the molecular weight between the two polymers. These findings are in good agreement with that detected by Maher et al., who found that the type of polymer used (hydroxypropyl methyl cellulose and carboxymethyl cellulose) had a significant effect on the tensile strength of the prepared films [55].

Moreover, it was noticed that increasing the polymer concentration (X_2_) significantly increased the tensile strength values of the prepared OFDFs. This could have been because of the formation of a densely packed network of the used polymer chains at higher concentration, leading to formation of a stronger matrix. Bharti et al. stated similar findings, where they found that the tensile strength of the prepared films was increased by increasing the film’s former concentration (hydroxypropyl methyl cellulose polymer) [33].

On the other hand, the tensile strength of PX OFDFs prepared with different plasticizer types (X_3_) was increased in this order: PG > PEG 400 > glycerol. It can be noticed that OFDFs prepared with glycerol as a plasticizer showed the lowest tensile strength values. This could be because of the high hygroscopicity of glycerol, which causes humidity absorption and consequently gives softness to the prepared films and decreases the tensile strength values [56].

##### Percentage Elongation

OFDFs should possess a large elongation percentage, in order to exhibit the desired flexibility and stretchability, which is important for facile handling and application of the film to the buccal cavity [15]. The percentage elongation values for all the prepared OFDFs were found to be from 6.03 ± 0.45% to 53.08 ± 1.28%, as displayed in Table 3. The following equation was used to analyze the percentage elongation values:

Percent Elongation = 26.29 + 1.41 × X_1_ + 0.44 × X_2_ + 22.68 × X_3_ [1] − 12.57 × X_3_ [2] (10)

where X_1_ is the polymer type, X_2_ is the polymer concentration, and X_3_ is the plasticizer type. X_3_ [1] represents the first plasticizer type (glycerol) and X_3_ [2] represents the second plasticizer type (propylene glycol).

Table 3 and Figure 2b show that the percentage elongation of the prepared OFDFs was significantly affected only by the plasticizer type (X_3_). Where, the percentage elongation of the OFDFs prepared with glycerol had the highest percentage elongation values.

The increase in the OFDFs elongation percentage can be attributed to the fact that glycerol replaces the intermolecular bonds present between polymer matrixes with hydrogen bonds created between polymer and glycerol molecules. This disruption and reconstruction of polymer molecular chains allows greater chain mobility, resulting in decreasing the rigidity and providing flexibility and stretching to the films [57]. These findings are in accordance with those stated by Junmahasathien et al., who reported that glycerol was the best plasticizer for increasing the elongation percentage of the prepared films, in comparison with other plasticizers [58].

##### Young’s Modulus

OFDFs should have low Young’s modulus values, to exhibit the desired elasticity; whereby, high values of Young’s modulus lead to the formation of stiff and brittle films [59]. All the prepared OFDFs showed Young’s modulus values from 8.09 ± 0.15 to 383.66 ± 11.06 Mpa, as displayed in Table 3. The following equation was used to analyze the Young’s modulus values:

Young’s modulus = 234.50 − 3.64 × X_1_ − 1.64 × X_2_ − 224.60 × X_3_ [1] + 151.74 × X_3_ [2] (11)

where X_1_ is the polymer type, X_2_ is the polymer concentration, and X_3_ is the plasticizer type. X_3_ [1] represents the first plasticizer type (glycerol) and X_3_ [2] represents the second plasticizer type (propylene glycol).

Table 3 and Figure 2c show that the plasticizer type (X_3_) was the only factor significantly impacting the Young’s modulus of the prepared OFDFs. Where, the OFDFs prepared using glycerol had the lowest Young’s modulus values.

This could be attributed to glycerol being capable of dispersing between the spaces of the polymer chains, reducing their intermolecular attraction and, hence, providing flexibility to the films [60].

#### 3.5.8. In Vitro Disintegration Time

Disintegration time is a very important parameter for OFDFs, to indicate the onset of drug action. A low value of disintegration time, allows a faster release and absorption of the loaded drug through the buccal mucosa. The mean disintegration time of all the prepared OFDFs ranged from 17.09 ± 1.30 to 160.06 ± 4.20 s, as displayed in Table 3. The following equation was used to analyze disintegration time values:

Disintegration time = 75.57 + 45.41 × X_1_ + 9.98 × X_2_ − 11.92 × X_3_ [1] − 3.36 × X_3_ [2] (12)

where X_1_ is the polymer type, X_2_ is the polymer concentration, and X_3_ is the plasticizer type. X_3_ [1] represents the first plasticizer type (glycerol) and X_3_ [2] represents the second plasticizer type (propylene glycol).

**Figure 3:**
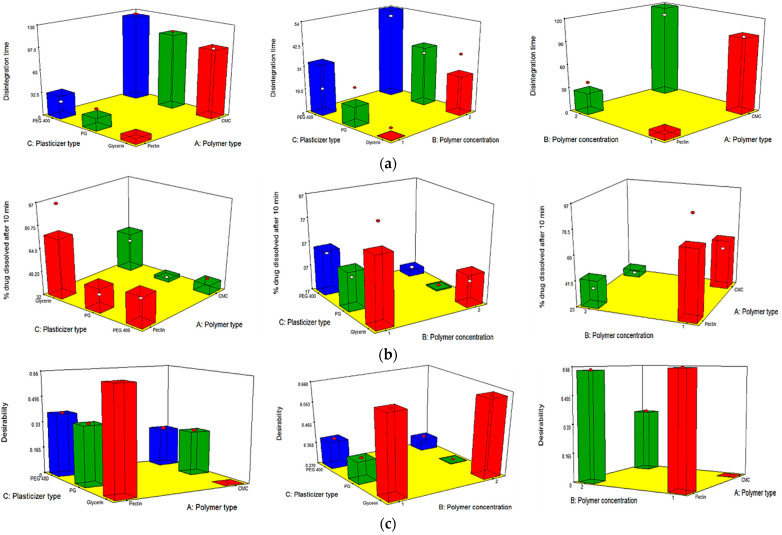
Response 3-D plots for the effect of polymer type (X_1_), polymer concentration (X_2_), and plasticizer type (X_3_) on (**a**) disintegration time, (**b**) % PX dissolved after 10 min, and (**c**) desirability of OFDFs loaded with PX nanosuspension.

Table 3 and Figure 3a show that all the independent variables (X_1_, X_2_ and X_3_) had a significant effect on the disintegration time of the prepared OFDFs. Disintegration time values were significantly changed when changing the type of polymer used (X_1_). Where, OFDFs prepared using pectin polymer, showed significantly lower disintegration time values than those prepared with CMC polymer. This could be because of the difference in the nature of the used polymers; wherein, pectin is more hydrophilic than CMC [61]. Thus, OFDFs prepared with pectin had a faster hydration, and hence faster disintegration, than those prepared with CMC.

Additionally, it is worth noting that increasing the polymer concentration (X_2_) resulted in a significant increase in the disintegration time of the prepared OFDFs. This could have been because increasing polymer concentration leads a need for more fluids to wet the films and increasing the film thickness, which retarded the penetration of water. Moreover, Shen et al., who formulated fast-dissolving films loaded with herpetrione nanoparticles, stated that the disintegration time of the formulated films increased with increasing HPMC concentration. He attributed this to the fact that increasing the HPMC concentration increases water viscosity when the film comes into contact with it, which inhibited its intake and retarded the film disintegration [20].

On the other hand, changing the plasticizer type (X_3_) significantly influenced the disintegration time of the prepared OFDFs. Where, OFDFs containing glycerol as a plasticizer were found to have a lower disintegration time than OFDFs prepared with PG and PEG 400. These results were in harmony with those stated by Singh et al., who prepared oral films loaded with desloratadine [50].

#### 3.5.9. In Vitro Dissolution Studies

**Figure 4:**
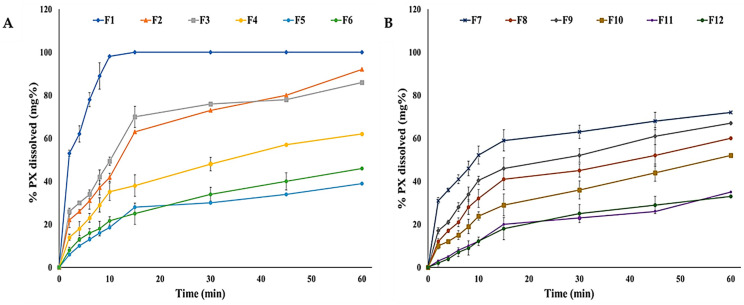
Dissolution profiles of the prepared (**A**) pectin and (**B**) CMC OFDFs loaded with PX nanosuspension.

For OFDFs, time is an important factor, because the loaded drug should be dissolved within a minute. Figure 4 illustrates the dissolution profiles of all the prepared OFDFs; where, the % PX dissolved after 10 min from all the prepared OFDFs was found to be from 12.14 ± 0.08% to 96.02 ± 3.46%, as displayed in Table 3. The following equation was used to analyze the % PX dissolved after 10 min:

% PX dissolved after 10 min = 36.27 − 7.47 × X_1_ − 15.69 × X_2_ + 15.53 × X_3_ [1] − 10.15 × X_3_ [2] (13)

where X_1_ is the polymer type, X_2_ is the polymer concentration, and X_3_ is the plasticizer type. X_3_ [1] represents the first plasticizer type (glycerol) and X_3_ [2] represents the second plasticizer type (propylene glycol).

Table 3 and Figure 3b show that all the independent variables (X_1_, X_2_, and X_3_) had a significant influence on the % PX dissolved after 10 min from the prepared OFDFs.

The polymer type (X_1_) had a significant influence on the % PX dissolved after 10 min_._ Where, OFDFs prepared by pectin polymer showed higher % PX dissolved after 10 min upon comparison with CMC-based OFDFs. This could have been because pectin has a more hydrophilic nature than CMC, resulting in faster hydration of pectin OFDFs, as explained previously [61]. Moreover, this could be because of the ionization of pectin at pH 6.8 (pH of the utilized dissolution medium), which is >pKa value of pectin (3.5) [62]. The ionization of pectin resulted in the presence of negative charges on the pectin backbone. Thus, the pectin polymer was uncoiled in the form of an extended structure, because of the negative charge repulsion, and the diffusion of positive charges within the pectin matrix generated an extra difference in the osmotic pressure across the matrix, which caused a higher water uptake. Hence, the pectin polymer swelled, resulting in drug diffusion from the films at a higher rate [63].

In addition, the statistical analyses clarified that polymer concentration (X_2_) had a significant impact on % PX dissolved after 10 min. Where, higher % PX dissolved after 10 min was shown in OFDFs with lower polymer concentration. Shaikh et al. reported similar findings, where they found that using a low polymer concentration led to needing a lower amount of water to dissolve the film and leading to faster drug release [64].

On the other hand, plasticizer type (X_3_) had a significant impact on %PX dissolved after 10 min. Where, OFDFs contained glycerol as a plasticizer had a higher %PX dissolved than those films contained PG or PEG 400. As explained earlier, this could be attributed to the high glycerol hygroscopicity, which led to more humidity absorption. This resulted in increasing the film hydrophilic character and increasing the %PX dissolved [56].

### 3.6. Selection of the Optimized OFDF Loaded with PX Nanosuspension

To identify the optimal OFDF, it was nearly impossible to fulfill all the desired responses at the same time, because the optimum condition fulfilled for one response could adversely affect other responses [22]. However, the desirability function combined all the desired responses in one variable, in order to determine the optimum levels of the examined factors [31]. Figure 3c shows that the highest desirability value was 0.659 for the optimized PX OFDF (F1) containing pectin polymer with a concentration of 1% *w/v* and glycerol as a plasticizer. This optimized OFDF (F1) collectively showed the maximal tensile strength, elongation %, % PX dissolved after 10 min, and minimal Young’s modulus and disintegration time. Where, OFDF (F1) showed a tensile strength of 3.89 ± 0.19 Mpa, elongation % of 53.08 ± 1.28, Young’s modulus of 8.12 ± 0.13 Mpa, disintegration time of 17.09 ± 1.30 s, and 96.02 ± 3.46% PX dissolved after 10 min. Upon comparing the observed and predicted values, they were found to be very similar; as shown in Table 2. Consequently, the optimized OFDF (F1) was chosen for further investigation.

### 3.7. Characterization of the Optimized OFDF Loaded with PX Nanosuspension

#### 3.7.1. Re-Dispersion of PX Nanoparticles from the Optimized OFDF

There was no significant difference between the PS of PX nanosuspension (217.09 ± 4.18 nm) and PS measures after re-dispersion of the optimized OFDF (231.88 ± 3.50 nm) (*p* < 0.05). This slight increase in PS could be attributed to coating the embedded nanoparticles with the polymeric matrix (pectin) and the plasticizer (glycerol) used in preparing the film. These results indicate the stability of the PX nanoparticles within the polymeric matrix [15].

#### 3.7.2. Comparative Dissolution Study of the Optimized OFDF (F1), Pure Drug, and the Market Tablet

**Figure 5:**
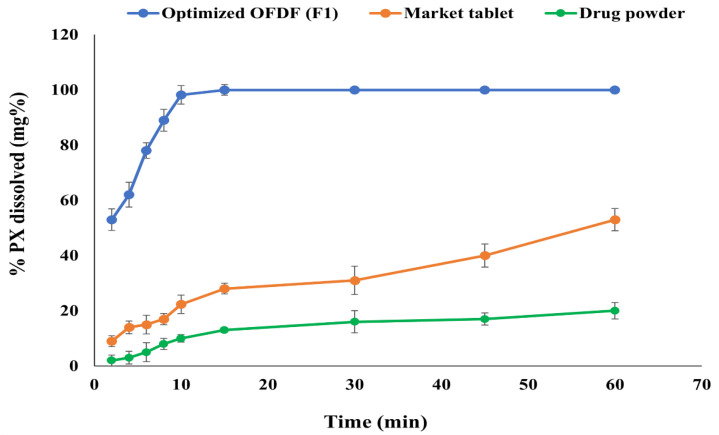
Dissolution profiles of the optimized OFDF loaded with PX nanosuspension (F1), PX pure powder, and the market tablet.

The optimized OFDF (F1) showed a significant increase in PX dissolution rate and extent in comparison with the PX dissolution from pure drug powder and the market tablet, with *f*_2_ values of 6 and 11, respectively. Figure 5 illustrates that 96.02% (more than 75%) PX was dissolved within just 10 min from the optimized OFDF (F1), compared with 10.04 and 26.37% from pure PX powder and the market tablet, respectively. The extent of dissolution of the optimized OFDF (F1) after 10 min was increased by more than 9.5 and 3.6 fold compared to the drug released from the pure drug and the market tablet, respectively. Additionally, the release T_50%_ of the optimized OFDF (F1) was 3.96 min, while the release T_50%_ of PX pure powder and the market tablet were 112.24 min and 54.16 min, respectively.

This could be attributed to the addition of tween 80, which enhanced the drug release from the optimized OFDF (F1), due to aiding in the acceleration of the disintegration of the film and releasing the incorporated drug more rapidly [15].

Another important reason for enhancement of the PX dissolution from the optimized OFDF was the formulation of the loaded drug as nanosized drug particles, which increased the drug solubility due to embedding the nanosized PX particles in the hydrophilic matrix. Moreover, the presence of the drug at nano-size could also have decreased the diffusion layer thickness, increasing the concentration gradient, which consequently increased the drug dissolution rate from the optimized film [65].

The low PX release from the pure PX powder could be attributed to the poor dissolution rate of paroxetine [6].

#### 3.7.3. Ex Vivo Permeation Studies

Many permeability studies have revealed the importance of choosing an appropriate mucosal membrane; where, the buccal mucosa of many experimental animals, such as rabbits and rats, is entirely covered with keratin [66]. On the other hand, chicken buccal mucosa (pouch) is considered the best alternative, because it resembles the human non-keratinized and thin oral lining mucosa [67].

**Figure 6:**
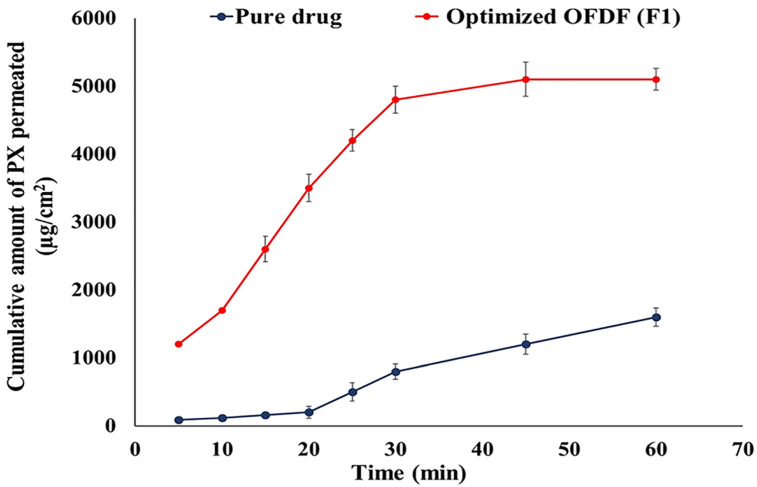
Permeation profiles of PX from the optimized OFDF (F1) and the pure drug through the chicken buccal mucosa.

The PX permeation from the optimized OFDF (F1) and PX pure powder through the freshly excised chicken buccal mucosa (pouch) was examined. HPLC analysis showed a linearity coefficient, LLOQ, and accuracy range of 0.9994, 10 μg/mL, and 100% ± 10, respectively. The optimized OFDF (F1) showed a significant increase in PX permeation rate and extent when compared to PX pure powder, as illustrated in Figure 6. The flux (J) values were detected and there was a significant difference (*p* value < 0.001) between the optimized OFDF (F1) (85.00 μg/h/cm^2^) when compared with PX pure powder (26.66 μg/h/cm^2^). The enhancement ratio (ER) value of the optimized OFDF (F1) was also estimated and was found to be 3.18, reflecting a more than three-fold increase in PX permeation through the buccal mucosa.

These results could be attributed to coating the loaded PX NPs with hydrophilic polymer, which enhanced the drug solubility and increased the surface area in contact with the mucosal membrane surface, resulting in increased permeability [15]. These results could also be attributed to the incorporation of tween 80 into the optimized film. Whereby, this surfactant provided an elastic effect that loosened or fluidized the mucosal membrane lipid bilayer and made the drug capable of squeezing into deeper layers of the biological membrane, leading to the enhancement of drug permeability through the buccal mucosa [31]. Regarding the mechanism of PX transport across the buccal mucosal membrane, the uptake process was concentration-dependent, via simple diffusion [67].

#### 3.7.4. Stability Study

Table 4 illustrates that there were no significant changes (*p* > 0.05) concerning all the investigated parameters of the optimized OFDF (F1) upon storage under the applied stability conditions.

### 3.8. In Vivo Clinical Studies

#### 3.8.1. In Situ Disintegration Time and Palatability Studies

The recorded results for texture evaluation showed that 87.5% of the enrolled volunteers stated that the optimized OFDF (F1) was flexible, easy to handle, and non-sticky.

The enrolled volunteers declared that the mean in situ disintegration time of the optimized OFDF (F1) was less than 1 min (14.84 ± 2.15 s), which is similar to the previously recorded in vitro disintegration time of the optimized OFDF (17.09 ± 1.30 s). This fast-disintegration could have been due to citric acid’s incorporation in the OFDF, which stimulated the saliva secretion in the buccal cavity, promoting rapid disintegration of the OFDF [51].

Regarding the results of the taste evaluation, 100% of the volunteers stated that PX pure powder was very bitter. While, in the case of the optimized OFDF (F1), 12.5% of the volunteers stated an acceptable taste, 25% stated pleasant taste, and 62.5% stated a very pleasant taste. These findings represent a success in masking PX’s bitter taste by incorporating sucralose as a sweetening agent [52]. Moreover, approximately 87.5% of the volunteers reported a mouth refreshment feeling. This feeling can be attributed to the incorporation of menthol in the OFDF [53].

All the enrolled volunteers also stated that the aftertaste of the optimized OFDF (F1) was significantly improved when compared to PX pure powder. Overall, these findings indicate that the optimized OFDF has the desired properties to be an easily handled and palatable fast-dissolving film.

#### 3.8.2. Pharmacokinetic Parameters of PX in Healthy Human Volunteer s

##### LC-MS/MS Method for Detection of Paroxetine in Human Plasma

No significant interference with paroxetine or Paroxetine-6D maleate (IS) was noticed in the chromatographed human plasma utilized in the preparation of quality control samples and calibration standards. The retention times of paroxetine and Paroxetine-6D maleate were 1.6 and 1.7 min, respectively. The linear relationship between PX concentrations and peak area ratio of PX/Paroxetine-6D maleate exhibited a linearity coefficient equal to 0.995, and the LLOQ was 1 ng/mL.

##### Estimation of Bioequivalence

**Figure 7:**
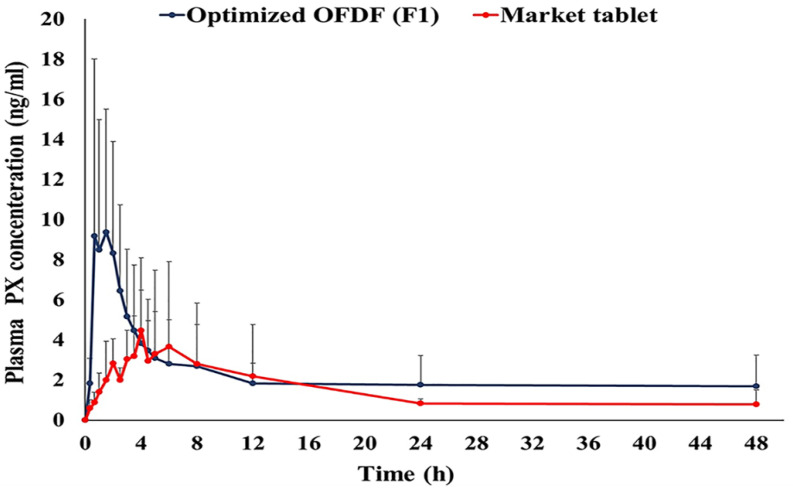
Mean paroxetine plasma concentration time curve after administration of the optimized OFDF (F1) and the market tablet to six healthy human volunteers.

All the volunteers tolerated the procedures executed in this study and the investigated drug well. Figure 7 illustrates PX mean plasma concentration–time profiles following oral administration of both the treatments. The PX pharmacokinetic parameters determined for the two treatments are shown in Table 5.

##### Statistical Analysis of Paroxetine Pharmacokinetic Parameters

It was noticed that the optimized OFDF (F1) had a significant increase in C_max_ (1.74 folds), AUC_0–48_ (1.56 folds), and AUC_0-∞_ (1.78 folds), when compared with the market tablet (*p*-value < 0.05). These results clarified that the extent of PX absorption from the optimized OFDF (F1) was significantly higher than the absorption from the market tablet. The optimized OFDF (F1) had a significantly lower T_max_ value when compared with the market tablet (*p*-value less than 0.05). This is might have been because of the rapid disintegration of the optimized OFDF and rapid dissolution of the PX in saliva, leading to fast absorption of PX through the buccal mucosa, and reaching higher plasma concentrations more rapidly [55]. The relative bioavailability of the optimized OFDF (F1) upon comparison with the market tablet was 178.43%. These findings fulfill the goal of this study, for enhancement of paroxetine bioavailability.

This increase in PX bioavailability can be credited to many reasons. First, the presence of the drug at nano-size within the OFDF, which led to increased drug solubility and dissolution rate, in comparison with the market product [16]. Second, the rapid disintegration of the optimized OFDF resulted in rapid absorption through the buccal mucosa and the prevention of large amounts of PX being metabolized in liver, achieving a higher bioavailability [53]. Third, the incorporation of tween 80 in the film formulation could have led to increased PX particle permeability at the absorption sites, which boosted the absorbed fraction of PX [17,21,27].

## 4. Conclusions

A PX nanosuspension, prepared using a solvent–antisolvent precipitation method, was successfully loaded into OFDFs prepared using a solvent casting method. The OFDFs loaded with PX nanosuspension represent a palatable and stable dosage method, which can be easily taken by pediatric, geriatric, and psychiatric patients. More than 90% of PX was dissolved within 10 min from the optimized OFDF, compared with 10.04 and 26.37% from the pure drug and the market tablet, respectively. A permeation study utilizing chicken buccal pouch revealed increasing drug permeation with the optimized OFDF, with a more than three-fold increase in permeation over the pure drug. Moreover, an in vivo bioavailability estimation in healthy human volunteers clarified that the optimized OFDF (F1) increased the PX bioavailability significantly more than the market tablet. Hence, the prepared OFDF can be considered a promising, convenient, and economical approach to boosting paroxetine bioavailability.

## Figures and Tables

**Table 1:** Experimental runs and independent variables of the full factorial experimental design for the prepared OFDFs loaded with PX nanosuspension.

Formulations	Factors (Independent Variables)		
	Polymer Type	Polymer Concentration (%*w/v*)	Plasticizer Type
F1	Pectin	1%	Glycerol
F2	Pectin	1%	PG
F3	Pectin	1%	PEG 400
F4	Pectin	2%	Glycerol
F5	Pectin	2%	PG
F6	Pectin	2%	PEG 400
F7	CMC	1%	Glycerol
F8	CMC	1%	PG
F9	CMC	1%	PEG 400
F10	CMC	2%	Glycerol
F11	CMC	2%	PG
F12	CMC	2%	PEG 400

Each prepared OFDF contained 250 mg plasticizer, 10 mg tween 80, 10 mg citric acid, 15 mg menthol, 50 mg sucralose, and distilled water to 10 mL.

**Table 2:** Output data of the factorial design and predicted and observed values for the optimized OFDF (F1).

Responses	Tensile Strength (Mpa)	% Elongation	Young’s Modulus (Mpa)	Disintegration Time (s)	% PX Dissolved after 10 Minutes
Minimum	1.04 ± 0.11	6.03 ± 0.45	8.09 ± 0.15	17.09 ± 1.30	12.14 ± 0.08
Maximum	15.5 ± 0.68	53.08 ± 1.28	383.66 ± 11.06	160.06 ± 4.20	96.02 ± 3.46
F value	17.21	20.73	15.61	79.52	11.31
*p*-value	0.0010	0.0006	0.0013	< 0.0001	0.0036
Adequate precision	13.39	9.83	18.01	22.99	10.88
Adjusted R^2^	0.855	0.877	0.842	0.966	0.789
Predicted R^2^	0.729	0.772	0.704	0.934	0.686
R^2^	0.908	0.922	0.899	0.978	0.896
Significant factors	X_1_, X_2_ and X_3_	X_3_	X_3_	X_1_, X_2_ and X_3_	X_1_, X_2_ and X_3_
Observed values of optimum OFDF (F1)	3.89	53.08	8.12	17.09	96.02
Predicted values of optimum OFDF (F1)	3.46	50.07	9.90	20.28	97.14

Data represented as mean ± SD (*n* = 3).

**Table 3:** Pharmaceutical evaluation and measured responses of the prepared oral fast-dissolving films loaded with PX nanosuspension.

	Average Weight (mg)	Film Thickness (mm)	Folding Endurance	Content Uniformity (%)	pH	Moisture Loss %	Moisture Absorption %	Tensile Strength (Mpa)	Percentage Elongation	Young’s Modulus (Mpa)	Disintegration Time (s)	% PX Dissolved after 10 Minutes
F1	38.02 ± 1.45	0.11 ± 0.02	>300	96.68 ± 3.62	6.80 ± 0.17	1.08 ± 0.02	1.39 ± 0.11	3.89 ± 0.19	53.08 ± 1.28	8.12 ± 0.13	17.09 ± 1.30	96.02 ± 3.46
F2	40.17 ± 0.98	0.13 ± 0.04	>300	93.14 ± 4.24	6.92 ± 0.11	1.24 ± 0.03	1.06 ± 0.08	9.68 ± 0.12	11.31 ± 1.06	306.41 ± 12.73	26.14 ± 3.06	41.74 ± 3.08
F3	39.29 ± 2.37	0.13 ± 0.01	>300	92.35 ± 1.28	6.64 ± 0.25	0.93 ± 0.02	1.21 ± 0.10	6.24 ± 0.26	11.06 ± 1.00	383.66 ± 9.06	20.27 ± 2.00	49.36 ± 2.63
F4	52.03 ± 1.88	0.15 ± 0.03	>300	91.50 ± 4.68	7.00 ± 0.16	1.16 ± 0.04	3.04 ± 0.27	6.63 ± 0.34	46.07 ± 2.46	13.98 ± 1.41	38.04 ± 1.38	35.15 ± 1.70
F5	52.61 ± 2.67	0.20 ± 0.01	203 ± 8.40	91.27 ± 2.89	6.62 ± 0.32	1.04 ± 0.02	3.62 ± 0.30	15.5 ± 0.68	18.1 ± 1.88	378.95 ± 16.86	34.25 ± 2.01	18.5 ± 1.55
F6	50.12 ± 1.48	0.18 ± 0.03	187 ± 5.71	94.04 ± 5.94	6.83 ± 0.24	1.17 ± 0.05	2.98 ± 0.12	6.71 ± 0.15	9.69 ± 2.08	337.75 ± 11.28	50.18 ± 1.33	21.62 ± 2.91
F7	41.07 ± 0.17	0.16 ± 0.06	>300	90.66 ± 3.36	7.01 ± 0.40	1.20 ± 0.04	5.66 ± 0.20	1.04 ± 0.11	49.82 ± 1.36	9.44 ± 0.27	97.23 ± 5.40	52.14 ± 4.07
F8	36.86 ± 3.96	0.15 ± 0.02	237 ± 10.00	93.78 ± 2.06	6.57 ±0.24	1.14 ± 0.03	6.35 ± 0.41	6.33 ± 0.32	6.03 ± 1.05	497.75 ± 22.47	110.79 ± 4.51	32.03 ± 1.08
F9	40.33 ± 2.07	0.13 ± 0.06	>300	92.04 ± 2.31	6.90 ± 0.14	0.96 ± 0.01	6.09 ± 0.26	3.68 ± 0.18	23.84 ± 2.04	211.5 ± 13.20	127.04 ± 4.38	40.44 ± 2.59
F10	49.20 ± 1.43	0.22 ± 0.01	>300	90.13 ± 4.63	6.84 ± 0.36	1.00 ± 0.04	8.73 ± 0.38	1.83 ± 0.10	46.93 ± 3.02	8.09 ± 0.15	107.23 ± 2.46	23.87 ± 1.16
F11	52.20 ± 3.75	0.22 ± 0.04	198 ± 6.70	89.48 ± 1.09	6.78 ± 0.12	1.18 ± 0.06	7.22 ± 0.19	8.95 ± 0.28	19.46 ± 1.80	361.87 ± 20.89	123.51 ± 3.87	12.19 ± 1.64
F12	50.49 ± 2.03	0.23 ± 0.03	240 ± 12.00	90.29 ± 2.43	6.61 ± 0.38	0.99 ± 0.05	8.64 ± 0.42	6.19 ± 0.27	20.13 ± 1.07	296.52 ± 14.04	160.06 ± 4.20	12.14 ± 0.08

**Table 4:** Stability test parameters for the optimized OFDF (F1).

Optimized OFDF (F1)	Content Uniformity (%)	Tensile Strength (Mpa)	% Elongation	Young’s Modulus (Mpa)	Disintegration Time (s)	% PX Dissolved after 10 min
Freshly prepared	96.68 ± 3.62	3.89 ± 0.19	53.08 ±1.28	8.12 ± 0.13	17.09 ± 1.30	96.02 ± 3.46
After 3 months	95.70 ± 3.14	4.02 ± 0.25	48.34 ± 0.03	8.06 ± 0.32	15.24 ± 0.87	96.50 ± 1.78
After 6 months	93.89 ± 4.08	3.93 ± 0.12	48.29 ± 0.16	7.99 ± 0.30	20.33 ± 1.01	95.63 ± 2.44

**Table 5:** Drug pharmacokinetic parameters after the administration of the optimized OFDF (F1) compared to the market tablet.

Pharmacokinetics Parameter	Treatment (Mean ± SD)	
	Optimized OFDF (F1)	Market Tablet
C_max_ (ng/mL) ^a^	11.18 ± 7.86	6.44 ± 3.77
AUC_0–48_ (ng.h/mL) ^a^	108.92 ± 81.31	69.79 ± 52.92
AUC_0-∞_ (ng.h/mL) ^a^	165.07 ± 135.10	92.51 ± 67.35
t_max_ (h) ^a^	0.94 ± 0.54	3.08 ± 1.88
t_1/2_ (h) ^a^	22.54 ± 4.11	22.32 ± 4.81
K (l/h) ^a^	0.030 ± 0.01	0.030 ± 0.01
MRT ^a^	37.90 ± 7.29	34.06 ± 8.24
% Relative bioavailability (%RB)	178.43	-

^a^ Data are the mean values (*n* = 6) ± SD.
